# Effects of resilience and timing of adverse and adaptive experiences on interpersonal behavior: a transdiagnostic study in a clinical sample

**DOI:** 10.1038/s41598-023-44555-z

**Published:** 2023-10-24

**Authors:** Barbara B. Barton, Thomas Ehring, Matthias A. Reinhard, Stephan Goerigk, Torsten Wüstenberg, Richard Musil, Benedikt L. Amann, Andrea Jobst, Julia Dewald-Kaufmann, Frank Padberg

**Affiliations:** 1 Department of Psychiatry and Psychotherapy, LMU University Hospital, Munich, Germany; 2 Department of Psychology, LMU Munich, Munich, Germany; 3 Charlotte Fresenius Hochschule, Infanteriestrasse 11A, 80797 Munich, Germany; 4 Department of Psychological Methodology and Assessment, Ludwig-Maximilians-University Munich, Munich, Germany; 5 Department of Psychiatry and Psychotherapy, Charité Campus Mitte, Charité – Universitätsmedizin Berlin, Berlin, Germany; 6 Centre Fòrum Research Unit, Hospital Del Mar Research Institute, Barcelona, Spain; 7 Mental Health Institute, Hospital Del Mar, Barcelona, Spain; 8 Universitat Pompeu Fabra, Barcelona, Spain

**Keywords:** Risk factors, Psychology, Human behaviour

## Abstract

Adverse childhood experiences (ACE) have been linked to less prosocial behavior during social exclusion in vulnerable groups. However, little is known about the impact of the timing of ACE and the roles of protective factors. Therefore, this study investigated the association of the behavioral response to experimental partial social exclusion with adverse and adaptive experiences across age groups and resilience in clinical groups with persistent depressive disorder and borderline personality disorder, i.e., groups with high ACE, and in healthy controls (HC) (N = 140). Adverse and adaptive experiences during childhood, youth, and adulthood were assessed with the Traumatic Antecedents Questionnaire, and resilience was measured with the Connor Davidson Resilience Scale. A modified version of the Cyberball paradigm was used to assess the direct behavioral response to partial social exclusion. In patients, adverse events during youth (*B* = − 0.12, *p* = 0.016) and adulthood (*B* = − 0.14, *p* = 0.013) were negatively associated with prosocial behavior, whereas in the HC sample, adaptive experiences during youth were positively associated with prosocial behavior (*B* = 0.25, *p* = 0.041). Resilience did not mediate these effects. The findings indicate that critical events during youth may be particularly relevant for interpersonal dysfunction in adulthood.

## Introduction

Adverse childhood experiences (ACE), such as child abuse and neglect, can have a detrimental impact on mental health, including causing dysfunctional interpersonal behavior^1^. Furthermore, a previous study that used a modified version of the Cyberball paradigm, a virtual ball-tossing game, found that ACE measured by the Childhood Trauma Questionnaire (CTQ)^2^ were associated with less prosocial behavior in response to partial social exclusion^3^ (the modified Cyberball paradigm^4^ induces social exclusion by partially excluding the participant from the game, i.e., the participant is excluded by only one of the two co-players). However, the CTQ does not allow specific conclusions to be drawn regarding the age at which ACE have critical effects or the impact of adaptive events because it asks about maltreatment in childhood until the age of 18. Thus, a uniform conceptualization of ACE may be misleading because ACE are diverse in type and severity and may occur at different developmental stages from childhood to adulthood (e.g. Ref.^5^). Although ACE have been extensively studied in childhood, less is known about the impact of adversity explicitly during youth and adulthood^6^, even though it can also have detrimental effects on mental health when it occurs in those age groups^7^. Studies directly investigating the timing of adverse life events and their effects on psychopathology are scarce, and the results are mixed. For example, ACE at an early age may be particularly toxic because they may interrupt development of the brain and social skills^8^, which reduces the capacity to use the environment as a source of resilience^9^. When adversity occurs during the transition to school age, prosocial behavior may be particularly negatively affected^10^. However, unlike children, adolescents and adults have fully developed cognitive skills, so they may be aware of the adversity’s detrimental effect, potentially making its impact even worse^11^.

ACE increase the risk of developing various psychiatric disorders and are associated with an early onset, a more severe clinical course, and less response to psycho- and pharmacotherapy^12^. For example, patients with borderline personality disorder (BPD) and persistent depressive disorder (PDD) report a high prevalence of ACE measured with the CTQ that assesses adverse events up to the age of 18^13^. Although patients with BPD also report increased experiences such as violence during youth and adulthood^14^, there is a lack of data on the prevalence of adverse events during adolescence and adulthood in patients with PDD. Furthermore, studies often only evaluate the five subtypes of childhood maltreatment (emotional abuse, emotional neglect, physical abuse, physical neglect, and sexual abuse) but do not include other stressful life events, e.g., separation from parents, even though one third of adolescents with BPD report these types of ACE^15^.

It is important to note that not all individuals who experience maltreatment develop a psychiatric disorder, probably because of various resiliency factors^12^ and gene-environment interactions, neither of which are fully understood yet^16^. In addition, both BPD and PDD patients are characterized by interpersonal difficulties. Patients with BPD have difficulties maintaining relationships because of their emotional instability, anger outbursts, fear of abandonment, and changes in the way they perceive their significant others^17^. Patients with PDD have been found to show hostile-submissive behavior, which probably results in loneliness and a small social network^18,19^. In an earlier study, we found that compared with healthy controls (HC), patients with BPD and PDD show deviant behavior in reaction to partial social exclusion induced by the modified Cyberball paradigm^20^. Although HC immediately increased the number of ball tosses to the player who was excluding them, which was interpreted as prosocial behavior, PDD patients showed no such change in behavior. In BPD patients, this immediate behavioral reaction was not significantly different from that in HC or PDD^20^.

The pathways explaining the relationship between adverse life events and interpersonal difficulties are not well understood. One possible pathway may be resilience, i.e., the ability to adapt to adverse events such as extreme stress caused by interpersonal problems, trauma, or threats^21^. Studies have shown that self-reported childhood adversity is associated with less resilience in adulthood^22,23^ and that a higher amount of experienced family stress in the past might weaken the growth of resilience^24^. However, Nishimi, Choi^22^ found no association between the developmental period in which childhood adversity occurred and resilience in adulthood. Furthermore, positive interpersonal experiences, such as parental care and peer relationships, can enhance resilience in individuals after severe physical and sexual abuse^25^. In fact, when predicting resilience, adaptive life events, e.g., support from friends, might even have more impact than adversity^26^. Resilience is positively correlated with more intact social functioning^27^ and negatively correlated with emotional and behavioral problems^28^. Studies in adolescents showed that childhood adversity led to more behavioral and emotional problems as assessed by a questionnaire (e.g., on prosocial behavior) and that the relationship was mediated by resilience, i.e., childhood adversity leads to less resilience, which leads to more behavioral and emotional problems^29^. Even though BPD is discussed as the consequence of a lack of resilience^30^ and depression has been linked to lower resilience^31^, research is lacking, and no study to date has explicitly reported on the resilience of patients with PDD.

The aim of this study was to analyze associations between adverse and adaptive life events during childhood, youth, and adulthood, resilience, and the immediate behavioral reaction to partial social exclusion during the modified Cyberball paradigm in patients with BPD and PDD, two disorders typically related to ACE, and HC. In addition, we compared resilience in the clinical sample and the HC and tested the effect of resilience on the relationship between adverse and adaptive events during childhood, youth, and adulthood and the behavioral response to partial social exclusion during Cyberball. On the basis of previous studies, we hypothesized that (a) adverse life events are associated with less prosocial behavior, i.e., lower rates of tossing the ball to the excluder, and lower resilience scores, whereas adaptive life events are related to higher rates of tossing the ball to the excluder and higher resilience scores; (b) resilience is significantly lower in patients with BPD and PDD than in HC; (c) resilience mediates the effect of adverse and adaptive life events on the immediate behavioral response to partial social exclusion during Cyberball; and (d) patterns in these associations differ between the clinical sample with an ACE history and HC.

## Results

### Sample characteristics

Compared with the HC, in each developmental phase the patient sample had significantly higher TAQ scores for adverse events, significantly lower TAQ scores for adaptive events, and significantly lower CD-RISC-10 scores (see Table 1). Results were the same when patients with BPD were compared with HC_BPD_ and patients with PDD, with HC_PDD_. Scores for adverse events overall and during childhood and youth were significantly higher in patients with BPD than in those with PDD, and resilience was significantly higher in patients with PDD than in those with BPD. However, none of the differences between patient groups was still significant after false discovery rate (FDR) correction. HC_BPD_ and HC_PDD_ did not differ on any of the self-report scales (see Table 1).

**Table 1:** Means, standard deviations, and one-way analysis of variance of study variables in patients with persistent depressive disorder (n = 34) and borderline personality disorder (n = 39) and healthy controls (n = 70).

	P	HC	BPD	PDD	HC_BPD_	HC_PDD_	one-way ANOVA	Planned Contrasts				
								Patient vs. HC	BPD vs. HC_BPD_	PDD vs. HC_PDD_	PDD vs. BPD	HC_BPD_ vs. HC_PDD_
TAQ_N_	4.36 (1.78)	2.02 (1.26)	4.85 (1.68)	3.81 (1.75)	1.79 (0.93)	2.27 (1.52)	*F*(3.69) = 34.73, ***p* < 0.001, *p***_***FDR***_** < 0.001**	*t*(108.88) = − 8.80, ***p* < 0.001**, ***p***_***FDR***_** < 0.001** *r* = 0.64	*t*(52.62) = − 9.44, ***p* < 0.001**, ***p***_***FDR***_** < 0.001** *r* = 0.79	*t*(59.53) = − 3.76 ***p* < 0.001**,*** p***_***FDR***_** < 0.001** *r* = 0.44	*t*(62.33) = 2.44, ***p* = 0.017**, *p*_*FDR*_ = 0.062 *r* = 0.30	*t*(52.06) = 1.54, *p* = 0.129,* p*_*FDR*_ = 0.462 *r* = 0.21
TAQ_NC_	1.32 (0.77)	0.53 (0.46)	1.50 (0.78)	1.11 (0.72)	0.50 (0.41)	0.55 (0.51)	*F*(3,70) = 19.06, ***p* < 0.001, *p***_***FDR***_** < 0.001**	*t*(104.39) = − 7.24, ***p* < 0.001**,*** p***_***FDR***_** < 0.001** *r* = 0.58	*t*(51.26) = − 6.73, ***p* < 0.001**, ***p***_***FDR***_** < 0.001** *r* = 0.68	*t*(53.26) = − 3.60, ***p* < 0.001**,*** p***_***FDR***_** < 0.001** *r* = 0.44	*t*(63.83) = 2.09, ***p* = 0.041**,* p*_*FDR*_ = 0.090 *r* = 0.25	*t*(63.48) = 0.43, *p* = 0.669,* p*_*FDR*_ = 0.867 *r* = 0.05
TAQ_NY_	1.54 (0.67)	0.67 (0.48)	1.73 (0.60)	1.32 (0.70)	0.59 (0.35)	0.76 (0.59)	*F*(3,69) = 35.55, ***p* < 0.001, *p***_***FDR***_** < 0.001**	*t*(105) = − 8.52, ***p* < 0.001**,*** p***_***FDR***_** < 0.001** *r* = 0.64	*t*(54.83) = − 9.78, ***p* < 0.001**, ***p***_***FDR***_** < 0.001** *r* = 0.80	*t*(58.74) = − 3.46, ***p* < 0.001**, ***p***_***FDR***_** < 0.001** *r* = 0.41	*t*(59.26) = 2.56, ***p* = 0.013**, *p*_*FDR*_ = 0.062 *r* = 0.32	*t*(51.35) = 1.40, *p* = 0.168,* p*_*FDR*_ = 0.462 *r* = 0.19
TAQ_NA_	1.50 (0.57)	0.81 (0.48)	1.61 (0.60)	1.38 (0.51)	0.70 (0.37)	0.92 (0.56)	*F*(3,132) = 22.75, ***p* < 0.001, *p***_***FDR***_** < 0.001**	*t*(132) = − 7.71, ***p* < 0.001**,*** p***_***FDR***_** < 0.001** *r* = 0.56	*t*(132) = − 7.47, ***p* < 0.001**, ***p***_***FDR***_** < 0.001** *r* = 0.55	*t*(132) = − 3.53, ***p* < 0.001**,*** p***_***FDR***_** < 0.001** *r* = 0.29	*t*(132) = 1.86, *p* = 0.065,* p*_*FDR*_ = 0.102 *r* = 0.16	*t*(132) = 1.84, *p* = 0.069,* p*_*FDR*_ = 0.380 *r* = 0.16
TAQ_P_	6.09 (2.15)	8.47 (0.63)	5.62 (2.29)	6.62 (1.87)	8.54 (0.38)	8.39 (0.82)	*F*(3,61) = 27.67, ***p* < 0.001, *p***_***FDR***_** < 0.001**	*t*(74.97) = 4.69, ***p* < 0.001**,*** p***_***FDR***_** < 0.001** *r* = 0.48	*t*(35.83) = 2.93, ***p* < 0.001**, ***p***_***FDR***_** < 0.001** *r* = 0.44	*t*(40.56) = 4.84, ***p* < 0.001**, ***p***_***FDR***_** < 0.001** *r* = 0.61	*t*(63.57) = − 1.97, *p* = 0.054,* p*_*FDR*_ = 0.099 *r* = 0.24	*t*(44.42) = − 1.01, *p* = 0.317,* p*_*FDR*_ = 0.581 *r* = 0.15
TAQ_PC_	1.99 (1.02)	2.83 (0.26)	1.85 (0.97)	2.16 (1.06)	2.84 (0.20)	2.82 (0.32)	*F*(3,63) = 15.16, ***p* < 0.001,* p***_***FDR***_** < 0.001**	*t*(69.21) = 6.38, ***p* < 0.001**,*** p***_***FDR***_** < 0.001** *r* = 0.61	*t*(36.88) = 3.37, ***p* = 0.002**, ***p***_***FDR***_** < 0.001** *r* = 0.49	*t*(34.95) = 3.37, ***p* = 0.002**,*** p***_***FDR* =**_** 0.002** *r* = 0.50	*t*(61.25) = − 1.21, *p* = 0.230, *p*_*FDR*_ = 0.253 *r* = 0.15	*t*(53.78) = − 0.27, *p* = 0.792,* p*_*FDR*_ = 0.871 *r* = 0.04
TAQ_PY_	2.00 (0.97)	2.79 (0.26)	1.81 (0.97)	2.23 (0.93)	2.81 (0.20)	2.76 (0.32)	*F*(3,63) = 15.24, ***p* < 0.001, *p***_***FDR***_** < 0.001**	*t*(73.12) = 6.34, ***p* < 0.001**,*** p***_***FDR***_** < 0.001** *r* = 0.60	*t*(36.82) = 6.03, ***p* < 0.001**, ***p***_***FDR***_** < 0.001** *r* = 0.70	*t*(36.56) = 3.02, ***p* = 0.005**,*** p***_***FDR* =**_** 0.006** *r* = 0.45	*t*(63.52) = − 1.79, *p* = 0.079,* p*_*FDR*_ = 0.109 *r* = 0.22	*t*(52.89) = − 0.84, *p* = 0.403,* p*_*FDR*_ = 0.633 *r* = 0.15
TAQ_PA_	2.09 (0.81)	2.85 (2.27)	1.96 (0.94)	2.24 (0.61)	2.89 (0.14)	2.81 (0.36)	*F*(3,60) = 21.31, ***p* < 0.001, *p***_***FDR***_** < 0.001**	*t*(72.58) = 7.36, ***p* < 0.001**, ***p***_***FDR***_** < 0.001** *r* = 0.65	*t*(35.56) = 5.83, ***p* < 0.001**, ***p***_***FDR***_** < 0.001** *r* = 0.70	*t*(47.75) = 4.50, ***p* < 0.001**, ***p***_***FDR***_** < 0.001** *r* = 0.55	*t*(59.10) = − 1.48, *p* = 0.144,* p*_*FDR*_ = 0.176 *r* = 0.19	*t*(42.71) = − 1.21, *p* = 0.234,* p*_*FDR*_ = 0.515 *r* = 0.18
TAQ item 43	2.62 (1.12)	1.43 (0.75)	3.00 (1.14)	2.19 (0.95)	1.39 (0.64)	1.47 (0.86)	*F*(3,132) = 23.86, ***p* < 0.001, *p***_***FDR***_** < 0.001**	*t*(132) = − 7.45, ***p* < 0.001**, ***p***_***FDR***_** < 0.001** *r* = 0.54	*t*(132) = 3.58, ***p* < 0.001**, ***p***_***FDR***_** < 0.001** *r* = 0.54	*t*(132) = − 3.19, ***p* = 0.002**,*** p***_***FDR* =**_** 0.002** *r* = 0.27	*t*(132) = − 7.44, ***p* < 0.001**, ***p***_***FDR***_** < 0.001** *r* = 0.30	*t*(132) = 0.37, *p* = 0.709, *p*_*FDR*_ = 0.087 *r* = 0.03
CD-RISC	16.17 (5.92)	29.60 (6.08)	14.75 (5.97)	17.71 (5.54)	30.86 (5.35)	28.26 (6.60)	*F*(3,135) = 63.04, ***p* < 0.001, *p***_***FDR***_** < 0.001**	*t*(136) = 13.35, ***p* < 0.001**, ***p***_***FDR***_** < 0.001** *r* = 0.75	*t*(135) = 11.62, ***p* < 0.001**, ***p***_***FDR***_** < 0.001** *r* = 0.71	*t*(135) = 7.34, ***p* < 0.001**,*** p***_***FDR***_** < 0.001** *r* = 0.53	*t*(135) = − 2.09, ***p* = 0.038,** *p*_*FDR*_ = 0.090 *r* = 0.18	*t*(135) = − 1.85, *p* = 0.067,* p*_*FDR*_ = 0.380 *r* = 0.16
PP	0.04 (0.27)	0.12 (0.25)	0.06 (0.25)	0.01 (0.29)	0.11 (0.23)	0.12 (0.28)	*F*(3,136) = 1.30, *p* = 0.279, *p*_*FDR*_ = 0.279	*t*(136) = 1.8, *p* = 0.074, *p*_*FDR*_ = 0.074 *r* = 0.15	*t*(136) = 0.81, *p* = 0.422, *p*_*FDR*_ = 0.422 *r* = 0.07	*t*(136) = 1.73, *p* = 0.086,* p*_*FDR*_ = 0.086 *r* = 0.15	*t*(136) = 0.83, *p* = 0.411,* p*_*FDR*_ = 0.411 *r* = 0.07	*t*(136) = 0.14, *p* = 0.892,* p*_*FDR*_ = 0.892 *r* = 0.01

*ANOVA* analysis of variance, *BPD* borderline personality disorder, *CD-RISC-10* Connor Davidson Resilience Scale 10-item, *FDR* false discovery rate, *HC* healthy controls, *P* patients, *PDD* persistent depressive disorder, *PP* passing preference during partial social exclusion (higher PP scores indicate increased ball tosses towards the excluder^4,20^), *TAQ* Traumatic Antecedents Questionnaire, *TAQ*_*N*_ TAQ total score for negative life events, *TAQ*_*NA*_ TAQ score for adverse events during adulthood, *TAQ*_*NC*_ TAQ score for adverse events during childhood, *TAQ*_*NY*_ TAQ score for adverse events during youth, *TAQ*_*P*_ TAQ score for total score adaptive life events, *TAQ*_*PA*_ TAQ score for adaptive events during adulthood, *TAQ*_*PC*_ TAQ score for adaptive events during childhood, *TAQ*_*PY*_ TAQ score for adaptive events during youth.

*α* = 0.05; significant results are indicated in bold face.

The TAQ was completed by 35 patients with BPD, 31 patients with PDD, 36 HC_BPD_, and 33 HC_PDD_, and the CD-RISC-10 was completed by 36 patients with BPD, 33 patients with PDD, 36 HC_BPD_, and 34 HC_PDD_.

### Relationship between passing preference and adverse/adaptive experiences and resilience

In the patient sample, adverse events during youth and adulthood showed a significant inverse correlation with passing preference (PP); higher levels of resilience were negatively associated with PP; a younger age was associated with higher PP; and an older age was associated with higher resilience (Table 2). In the HC sample, adaptive events during youth were significantly associated with a higher PP, higher resilience scores were significantly associated with adaptive events overall and during adulthood and older age was significantly associated with adverse events in general and during youth and adulthood (see Table 3).

**Table 2:** Correlations between age, Traumatic Antecedents Questionnaire and Connor Davidson Resilience Scale 10-item scores, and passing preference during partial social exclusion in patients with persistent depressive disorder (n = 34) and borderline personality disorder (n = 36).

PP	PP	TAQ_N_	TAQ_NC_	TAQ_NY_	TAQ_NA_	TAQ_P_	TAQ_PC_	TAQ_PY_	TAQ_PA_	CD-RISC-10
	*r* [BCa*CI*]	*r* [BCa*CI*]	*r* [BCa*CI*]	*r* [BCa*CI*]	*r* [BCa*CI*]	*r* [BCa*CI*]	*r* [BCa*CI*]	*r [BCaCI]*	*r [BCaCI]*	*r [BCaCI]*
Age	** − 0.307** [− 0.546; − 0.056] *p* = 0.012	0.009 [− 0.262; 0.297] *p* = 0.945	0.008 [− 0.280; 0.324] *p* = 0.951	− 0.065 [− 0.335; 0.229] *p* = 0.602	0.095 [− 0.112; 0.325] *p* = 0.450	− 0.078 [− 0.288; 0.137] *p* = 0.533	− 0.147 [− 0.382; 0.113] *p* = 0.239	− 0.015 [− 0.260; 0.204] *p* = 0.905	− 0.004 [− 0.200; 0.198] *p* = 0.972	**0.256** [− 0.021; 0.508] *p* = 0.038
PP		− 0.214 [− 0.436; 0.057] *p* = 0.084	− 0.078 [− 0.324; 0.211] *p* = 0.535	** − 0.244** [− 0.471; 0.025] *p* = 0.048	** − 0.275** [− 0.486; − 0.024] *p* = 0.025	0.003 [− 0.224; 0.230] *p* = 0.981	0.067 [− 0.207; 0.327] *p* = 0.594	0.101 [− 0.136; 0.344] *p* = 0.419	− 0.198 [− 0.391; 0.007] *p* = 0.111	** − 0.269** [− 0.479; − 0.044] *p* = 0.029
NTAQ			**0.895** [0.826; 0.938] *p* < 0.001	**0.912** [0.843; 0.955] *p* < 0.001	**0.828** [0.736; 0.897] *p* < 0.001	** − 0.421** [− 0.601; − 0.241] *p* < 0.001	** − 0.342** [− 0.587; − 0.109] *p* = 0.005	** − 0.403** [− 0.602; − 0.180] *p* < 0.001	− 0.205 [− 0.425; 0.024] *p* = 0.098	** − 0.**151 [− 0.404; 0.096] *p* = 0.226
TAQ_NC_				**0.727** [0.543; 0.851] *p* < 0.001	**0.587** [0.398; 0.743] *p* < 0.001	** − 0.407** [− 0.577; − 0.243] *p* < 0.001	** − 0.397** [− 0.623; − 0.168] *p* < 0.001	** − 0.290** [− 0.502; − 0.061] *p* = 0.018	− 0.234 [− 0.430; − 0.052] *p* = 0.059	− 0.172 [− 0.426; 0.065] *p* = 0.167
TAQ_NY_					**0.678** [0.514; 0.795] *p* < 0.001	** − 0.351** [− 0.525; − 0.155] *p* = 0.004	** − 0.259** [− 0.514; 0.007] *p* = 0.035	** − 0.381** [− 0.570; − 0.163] *p* = 0.002	− 0.150 [− 0.369; 0.081] *p* = 0.230	− 0.141 [− 0.399; 0.095] *p* = 0.257
TAQ_NA_						** − 0.346** [− 0.553; − 0.135] *p* = 0.004	− 0.222 [− 0.454; 0.012] *p* = 0.074	** − 0.414** [− 0.602; − 0.196] *p* < 0.001	− 0.146 [− 0.401; 0.115] *p* = 0.242	− 0.070 [− 0.313; 0.161] *p* = 0.575
PTAQ							**0.787** [0.649; 0.874] *p* < 0.001	**0.850** [0.757; 0.907] *p* < 0.001	**0.650** [0.416; 0.789] *p* < 0.001	0.233 [− 0.056; 0.465] *p* = 0.060
TAQ_PC_								**0.528** [0.256; 0.734] *p* < 0.001	0.202 [− 0.056; 0.450] *p* = 0.104	0.203 [− 0.073; 0.459] *p* = 0.101
TAQ_PY_									**0.397** [0.118; 0.606] *p* < 0.001	0.175 [− 0.082; 0.377] *p* = 0.160
TAQ_PA_										0.155 [− 0.180; 0.415] p = 0.215

*CD-RISC-10* Connor Davidson Resilience Scale, *PP* passing preference during social exclusion (higher PP scores indicate increased ball tosses towards the excluder^4,20^), *TAQ* Traumatic Antecedents Questionnaire, *TAQ*_*N*_ total score negative life events, *TAQ*_*NA*_ adverse events during adulthood, *TAQ*_*NC*_ adverse events during childhood, *TAQ*_*NY*_ adverse events during youth, *TAQ*_*P*_ total score adaptive life events, *TAQ*_*PA*_ adaptive events during adulthood, *TAQ*_*PC*_ adaptive events during childhood, *TAQ*_*PY*_ adaptive events during youth.

*α* = 0.05; significant results are indicated in bold face.

The TAQ was completed by 35 patients with BPD, 31 patients with PDD, 36 HC_BPD_, and 33 HC_PDD_; the CD-RISC-10 was completed by 36 patients with BPD, 33 patients with PDD, 36 HC_BPD_, and 34 HC_PDD_.

**Table 3:** Correlations between age, Traumatic Antecedents Questionnaire and Connor Davidson Resilience Scale 10-item scores, and passing preference during partial social exclusion in the healthy control sample.

PP	PP	NTAQ	TAQ_NC_	TAQ_NY_	TAQ_NA_	PTAQ	TAQ_PC_	TAQ_PY_	TAQ_PA_	CD-RISC-10
	*r* [BCa*CI*]	*r* [BCa*CI*]	*r* [BCa*CI*]	*r* [BCa*CI*]	*r* [BCa*CI*]	*r* [BCa*CI*]	*r* [BCa*CI*]	*r [BCaCI]*	*r [BCaCI]*	*r [BCaCI]*
Age	0.183 [− 0.034; 0.380] *p* = 0.131	**0.260** [− 0.041; 0.547] *p* = 0.031	0.163 [− 0.105; 0.419] *p* = 0.180	**0.245** [− 0.056; 0.535] *p* = 0.042	**0.277** [− 0.021; 0.557] *p* = 0.021	0.125 [− 0.041; 0.316] *p* = 0.307	0.162 [− 0.023; 0.362] *p* = 0.183	0.087 [− 0.109; 0.299] *p* = 0.476	0.048 [− 0.158; 0.242] *p* = 0.693	− 0.011 [− 0.275; 0.260] *p* = 0.927
PP		0.022 [− 0.247; 0.247] *p* = 0.855	0.130 [− 0.166; 0.380] *p* = 0.288	− 0.045 [− 0.252; 0.163] *p* = 0.711	− 0.019 [− 0.259; 0.211] *p* = 0.879	0.230 [− 0.014; 0.440] *p* = 0.057	0.190 [− 0.017; 0.340] *p* = 0.118	**0.250** [− 0.020; 0.459] *p* = 0.038	0.107 [− 0.167; 0.302] *p* = 0.379	0.015 [− 0.195; 0.255] *p* = 0.900
NTAQ			**0.843** [0.766; 0.899] *p* < 0.001	**0.930** [0.887; 0.957] *p* < 0.001	**0.878** [0.788; 0.932] *p* < 0.001	− 0.097 [− 0.324; 0.114] *p* = 0.427	0.056 [− 0.142; 0.281] *p* = 0.647	− 0.207 [− 0.455; − 0.070] *p* = 0.088	− 0.007 [− 0.304; 0.120] *p* = 0.530	− 0.092 [− 0.408; 0.226] *p* = 0.454
TAQ_NC_				**0.698** [− 0.009; 0.073] *p* < 0.001	**0.554** [− 0.011; 0.099] *p* < 0.001	− 0.103 [0.002; 0.107] *p* = 0.399	− 0.025 [0.007; 0.111] *p* = *0.*836	− 0.143 [− 0.002; 0.117] *p* = 0.241	− 0.075 [− 0.340; 0.120] *p* = 0.541	− 0.188 [0.154; − 0.408] *p* = 0.122
TAQ_NY_					**0.764** [0.611; 0.862] *p* < 0.001	− 0.120 [− 0.362; 0.120] p = 0.326	0.038 [− 0.147; 0.275] *p* = 0.755	− 0.234 [− 0.534; 0.128] *p* = 0.053	− 0.086 [− 0.277; 0.094] *p* = 0.481	− 0.050 [− 0.346; 0.274] *p* = 0.683
TAQ_NA_						− 0.036 [− 0.309; 0.163] *p* = 0.722	0.132 [− 0.071; 0.311] *p* = 0.279	− 0.170 [− 0.421; 0.128] *p* = 0.162	− 0.044 [− 0.321; 0.132] *p* = 0.722	− 0.011 [− 0.304; 0.307] *p* = 0.928
PTAQ							**0.801** [0.470; 0.912] *p* < 0.001	0.**716** [0.634; 0.915] p < 0.001	**0.845** [0.579; 0.939] *p* < 0.001	**0.287** [− 0.019; 0.538] *p* = 0.017
TAQ_PC_								**0.307** [0.042; 0.536] *p* = 0.010	**0.593** [− 0.025; 0.821] *p* < 0.001	0.179 [− 0.049; 0.389] *p* = 0.141
TAQ_PY_									**0.389** [0.116; 0.667] *p* = *0.*001	0.151 [− 0.105; 0.410] *p* = 0.215
TAQ_PA_										**0.343** [0.085; 0.639] *p* = 0.004

*CD-RISC-10* Connor Davidson Resilience Scale, *PP* passing preference during social exclusion (higher PP scores indicate increased ball tosses towards the excluder^4,20^), *TAQ* Traumatic Antecedents Questionnaire, *TAQ*_*N*_ total score negative life events, *TAQ*_*NA*_ adverse events during adulthood, *TAQ*_*NC*_ adverse events during childhood, *TAQ*_*NY*_ adverse events during youth, *TAQ*_*P*_ total score adaptive life events, *TAQ*_*PA*_ adaptive events during adulthood, *TAQ*_*PC*_ adaptive events during childhood, *TAQ*_*PY*_ adaptive events during youth.

*α* = 0.05; significant results are indicated in bold face.

The TAQ was completed by 35 patients with BPD, 31 patients with PDD, 36 HC_BPD_, and 33 HC_PDD_; the CD-RISC-10 was completed by 36 patients with BPD, 33 patients with PDD, 36 HC_BPD_, and 34 HC_PDD_.

In both the patient and the HC samples, events during childhood, youth, and adulthood were positively correlated in adverse and adaptive event domains; the only exception was in the patient sample, in which adaptive events during childhood were not correlated with adaptive events during adulthood. In the patient sample, some adverse events were negatively correlated with adaptive events, but adverse events during adulthood were not correlated with adaptive events during childhood and adulthood; further, adaptive events during adulthood were not correlated with adverse events during childhood, youth and adulthood (Table 2). These relationships were not found in the HC group (Table 3). Scatterplots of the main findings are presented in the supplemental materials (see Fig. S1).

### Conditional process analyses

The conditional direct effects showed that in patients, but not in HC, adverse events overall (*B* = − 0.04, *p* = 0.031) and during youth (*B* = − 0.12, *p* = 0.016) and adulthood (*B* = − 0.14, *p* = 0.013) predicted reduced PP. In contrast, in HC, but not in patients, adaptive events during youth were associated with more PP towards the excluder (*B* = 0.25, *p* = 0.041). Group as a predictor of resilience was significant when adverse events overall (*B* = − 12.47, *p* < 0.001) and during childhood (*B* = − 13.19,* p* < 0.001), youth (*B* = − 12.33*, p* < 0.001), and adulthood (*B* = − 12.38, *p* < 0.001) were used as independent variables. Adaptive events overall (*B* = 4.65, *p* = 0.044) and during adulthood (*B* = 13.55, *p* = 0.011) were associated with higher resilience scores. When adaptive events during adulthood were used as an independent variable, group moderated the effect of adaptive events on resilience (*R*^*2*^ = 0.02,* F* (1,131) = 5.18, *p* = 0.024). Overall, resilience scores did not mediate the relationship between adverse and adaptive events and PP in any of the models. Furthermore, the index of moderated mediation was insignificant (see Table S1 in the supplemental materials).

## Discussion

This study found that BPD and PDD patients with a history of ACE were different from HC regarding the association of adverse and adaptive life events on the one hand with the immediate prosocial reaction to partial social exclusion, i.e. ball tosses to the excluder during Cyberball, and resilience on the other. These effects were not mediated by resilience. Because our modified Cyberball task represents only one, rather specific experimental paradigm of social exclusion, the results of this study have to be interpreted carefully and generalization is limited.

In patients, but not in HCs, we found that adverse events in youth and adulthood were significantly negatively associated with the PP towards the excluding player during Cyberball. Furthermore, the conditional process analyses showed significant direct effects, indicating that adverse events in general and especially during youth and adulthood led to a reduced PP towards the excluder in patients. Interestingly, in this study adverse events during childhood (age 7–12 years) were not associated with the PP during Cyberball. Previous studies focused mostly on the detrimental impact of childhood adversity and found that it negatively impacts mental health, including interpersonal functioning^1^. However, youth is also a highly vulnerable phase^32^, and adolescents—like adults—are fully aware when they experience harm, which may make the harm especially detrimental. In addition, studies suggest that different adverse events might have different effects during different developmental stages in life^33^. Thus, childhood adversity may have a great impact on the development of psychiatric disorders in general, whereas adverse events during youth and adulthood may especially affect interpersonal skills. Nevertheless, maltreatment in early school ages may also negatively impact social behavior and interaction^10^. Our results suggest that adverse events during youth and adulthood should receive more attention in research on risk factors for interpersonal dysfunction in adulthood. In the HC group, only adaptive experiences during youth were significantly associated with more prosocial reactions, i.e., ball tosses to the excluder during Cyberball. The conditional process analyses also showed a significant effect of adaptive events during youth on PP. Again, youth seems to be a very critical phase for shaping interpersonal behavior.

Adaptive events were correlated with resilience only in HC, where adaptive experiences overall and during adulthood were associated with higher resilience scores; this correlation is in line with a previous study^25^. However, unlike other studies, including one by our group, adverse events showed no correlation with resilience^23^; in our previous study we also used both the TAQ and CD-RISC, however, the latter in its 25 item version^34^. Further interpretation of the findings on resilience is limited not only by the small sample size, but also by the fact that resilience is multifaceted, meaning that other factors besides adverse and adaptive life experiences, such as socioeconomic status, may be involved in the development of resilience^23^.

A higher resilience score was significantly associated with reduced prosocial behavior in the patients, but there was no significant relationship between resilience and PP in the HCs. Our findings are in contrast to studies that found an association of higher resilience with lower levels of emotional and behavioral problems, including prosocial behavior^29^. The results in the HC sample are in accordance with those of Wright, Fopma-Loy^35^, who also found no association between resilience and interpersonal functioning. Resilience also did not mediate the effect of adverse or adaptive life events on the immediate behavioral response to partial social exclusion during Cyberball. A possible explanation for the missing association of resilience with adverse and adaptive events and the missing mediating effect is that self-reported resilience does not necessarily translate into behavior. A study in college students showed that when they faced a social stressor, the students’ stress reactions, such as heartrate, were not actually different in those who reported high resilience compared with those who reported low resilience^36^. Nevertheless, self-reported resilience might affect how we reappraise stressful situations, which could protect us or reduce the emotional consequences after being socially excluded. Finally, as Bonanno^37^ suggests, small effects should be expected when studying resilience because resilience and the behavioral consequences might be very dependent on how someone evaluates a situation.

To our knowledge, no previous studies have examined resilience in PDD; moreover, data on BPD also are limited. The resilience scores of PDD patients were comparable to those of BPD patients and significantly lower than those of HC. Without FDR correction, the resilience scores of PDD patients were significantly higher than those of BPD patients. This finding is in accordance with previous findings of reduced resilience in BPD^30^ and of a negative correlation of resilience with depression^31^.

In the patient and HC samples, adverse events in all developmental phases showed intercorrelations, which is in line with research showing that victimization is often followed by further victimization^38^. Furthermore, adverse interpersonal experiences might lead to interpersonal difficulties and attract dysfunctional relationships that may be abusive or violent. ACE may therefore translate into real-life behavior. Behavioral paradigms such as Cyberball might help to better understand this translation. However, it is important to note that Cyberball may provoke a rather specific form of social distress and that BPD patients in particular are very sensitive to all kinds of invalidation, as suggested in the biosocial model of Linehan^39^. Another possible explanation could be that victimization brings back memories of earlier victimization, which could bias self-reports. Similarly, the positive association between adaptive events in different developmental phases could also be due to a reappraisal bias. However, this hypothesis may have therapeutic implications in that encouraging patients to identify past adaptive events could encourage them to recognize or reappraise future events in a more adaptive way and consequently strengthen resilience. Furthermore, the missing link between adverse events during childhood and adaptive events during adulthood in the patient sample highlights that new positive experiences are possible even after experiencing ACE.

### Strengths and limitations

The strengths of this study include the use of actual behavioral data to investigate interpersonal behavior and the recruitment of age- and sex-matched samples to test our hypotheses. However, this study also has some limitations. First, cross-sectional data from mediation analyses always need to be interpreted with caution^40^. Second, the majority of data relied on self-report measures, which can always be influenced by reporting bias^41^. When assessing childhood adversity, one has to consider that retrospective data tell us only about adversity that is remembered and appraised as such^42^. Thus, we have to be careful when concluding from our study that objective adversities have the same effect on interpersonal behavior. Third, the sample size is rather small, and further interpretation of negative findings may be hampered by a considerable beta error. Last, the social stressor used in the study, i.e., partial social exclusion induced by a modified version of Cyberball, is very specific, which limits generalization of the findings to other interpersonal situations.

### Future research

Larger sample sizes are needed to replicate our findings and further explore the role of resilience. Furthermore, in addition to self-report measures, studies should include interviews to assess negative life experiences and resilience. Relevant in this context is that after a stressful event, adaptation is rather the norm than the exception, e.g., after childhood adversity and interpersonal trauma, only 10% to 25% of people do not achieve resilient functioning^43^. Although the HC in our study reported low rates of adverse events, to better understand the role of psychopathology on interpersonal behavior, future studies should include participants who did not develop a psychiatric disorder despite experiencing multiple adverse events. Moreover, future studies should focus on adaptive experiences in patients with BPD and PDD because this topic has been neglected in research so far but may predict symptom severity and quality of life^44^. More knowledge on the impact of adaptive experiences and resilience is useful for therapy because both can be actively worked on, e.g., by improving resilient coping techniques. A recent study showed that the relationship between polyvictimization and cybervictimization was mediated by resilience, suggesting that promoting resilience in individuals with negative life events could help to further decrease victimization^45^. In addition, our study showed a high prevalence of ACE in patients with BPD who also had post-traumatic stress disorder as a comorbid disorder, so therapeutic approaches should consider incorporating trauma-focused therapies into individualized treatment plans.

Because resilience is multidimensional, even among people with the same level of resilience, some may use resilience in one domain but not in another^46^, i.e., participants with the same level of resilience might show a different behavioral reaction to being socially excluded during Cyberball. Thus, different methods for inducing social exclusion are needed to further explore the mediating effect of resilience.

This study showed that patients with ACE have distinct patterns in the associations between adverse and adaptive life events, resilience, and their responses to social exclusion. Interestingly, adverse events during youth and adulthood but not during childhood were found to affect interpersonal behavior in response to social exclusion. Only in the HC were adaptive events during adulthood positively associated with resilience, and only in the patients was resilience negatively associated with prosocial behavior. We did not find a mediating effect of resilience on the relationship between adverse or adaptive life events and the immediate behavioral reaction to partial social exclusion during Cyberball. This study partially fills a gap in the research on resilience in patients with PDD and BPD and provides evidence for the unexplored pathways from adverse and adaptive events to interpersonal behavior. In addition, it shows the high prevalence of ACE in patients with BPD and PDD compared with HC and adds important knowledge to the current literature, where studies on the effects of adversity during different periods of life in male and female patients with BPD and PDD are scarce. Multiple pathways may be involved in the association between adverse and adaptive life events and the behavioral reaction to social exclusion, and future research should try to elucidate these pathways because they may be a prerequisite for developing mechanism-based interventions for patients with ACE.

## Methods

### Participants

A total of 140 participants with BPD (*n* = 36, 47.2% male, 80.6% inpatients) and PDD (*n* = 34, 55.9% male, 85.3% inpatients) and age- and sex-matched HC (HC_BPD_, *n* = 36; HC_PDD_, *n* = 34) were recruited at LMU University Hospital and through flyers. The SCID I and II interviews^47^ were used to assess BPD, and the DSM-5 criteria were used to assess PDD^17^. Patients with PDD were excluded if they fulfilled more than three criteria of BPD. All participants were assessed with the Beck Depression Inventory (BDI)^48^ and Borderline Symptom List 23 (BSL)^49^. Exclusion criteria for HC were psychotherapy or pharmacotherapy in the past 10 years, depressive symptoms (defined as a score ≥ 12 on the BDI), fulfilling criteria for any psychiatric disorder, and pregnancy. Patients with BPD were significantly younger than those with PDD (BPD,* M*_age_ = 28.83, *SD* = 9.22; PDD,* M*_age_ = 38.16, *SD* = 12.34; *p* ≤ 0.001); similarly, HC_BPD_ were significantly younger than their HC_PDD_ counterparts (HC_BPD_, *M*age = 29.04, *SD* = 9.07; HC_PDD_, *M*age = 38.23, *SD* = 12.22; *p* ≤ 0.001). BDI scores were not different between patients with BPD and those with PDD (BPD, *M*_*BDI*_ = 31.36, *SD* = 10.71; PDD, *M*_*BDI*_ = 25.52, *SD* = 11.26; *p* = 0.132), but scores on the BSL-23 were significantly higher in patients with BPD than in those with PDD (BPD, *M*_*BSL*_ = 2.00, *SD* = 0.87; PDD: *M*_*BSL*_ = 1.01, *SD* = 0.65; *p* < 0.001); in addition, scores on both the BDI and BSL-23 were significantly higher in both patient groups than in the matched HC (all *p*s < 0.001).

The most common comorbidities in patients with BPD were anxiety disorders (61.1%), post-traumatic stress disorder (41.7%), and any substance abuse in the previous year (44.4%). Furthermore, 47.2% of BPD patients reported a current depressive episode, 80.6% reported lifetime depressive episodes, and 38.9% also fulfilled the criteria of PDD. In patients with PDD, the most common comorbidities were any cluster C personality disorder (44.1%), any anxiety disorder (35.3%), and any substance abuse in the previous year (20.6%).

All participants gave written informed consent before participating in the study, which was approved by the ethics committee of the Faculty of Medicine at the Ludwig-Maximilians-University, Munich, Germany (registration number: 281-11). The study was registered retrospectively at the German Clinical Trials Register (DRKS-ID: DRKS00019821; registered on May 28, 2020) as part of its parent study (registration number: 713-15). The Good Clinical Practice guidelines and the ethical guidelines of the German Psychological Society, a German adaption of the ethical guidelines provided by the American Psychological Association (“Ethical Principles of Psychologists and Code of Conduct”), were followed. A more detailed description of the participants is provided in earlier publications^3,18,20^.

### TAQ

The TAQ^50^ is a 43-item self-report questionnaire that assesses adverse and adaptive experiences during childhood (age 7–12 years), youth (age 13–18 years), and adulthood (age > 18 years). The questionnaire also includes an additional question about how distressing it was to answer the questions. The TAQ measures 2 domains on adaptive experiences (competence and safety) as well as 8 domains evaluating adverse experiences (neglect, separation from primary caregiver(s), emotional abuse, physical abuse/assault, sexual abuse/assault, witnessing, other traumas, and exposure to drugs and alcohol). Items are rated on a Likert scale ranging from 0 to 3 and a fourth option, “don’t know”; the responses to some items are dichotomous (yes/no). The TAQ also includes inverted items. In this study, “don't know" was marked with an asterisk (*) and then entered as 0; ratings of 0, 1, and “no” were recorded as 0; ratings of 2 were left as such; and ratings of 3 or “yes” were recorded as 3. The mean of all non-zero scores was then calculated for each domain. Adverse and adaptive domains were summed for each developmental phase and for the whole life span. A Korean validation study that used the 37-item TAQ reported a Cronbach’s alpha for the TAQ of greater than 0.80^51^.

### CD-RISC-10

The CD-RISC-10^52,53^ is one of the most widely used unidimensional self-report questionnaires for assessing self-perceived resilience^54^ and is recommended for the assessment of trait resilience^55^. The scale defines resilience as the ability to cope with stress, adapt to change, function under pressure, and achieve goals in the face of obstacles. Patients rate items on a 5-point Likert scale from 0 (never true) to 4 (always true). The sum score ranges from 0 to 40, with higher scores indicating higher resilience capacity. The psychometrics of the German version of the CD-RISC-10 are satisfactory (Cronbach’s alpha > 0.80)^55^.

### Cyberball

The Cyberball paradigm is a virtual ball tossing game and was shown to be a valid experimental approach to induce social exclusion^56^. In its original version by Williams, Cheung^57^ the participant is completely excluded by two co-players and thus does not receive any ball tosses. In this study, we used a modified version, created by co-author T.W., that uses partial social exclusion because we were interested to evaluate how participants behaviorally reacted to social exclusion. The goal of the modified Cyberball version is to assess the participant’s actual behavioral response, i.e., to assess ball tossing behavior, which is labeled as PP. The participant is led to believe that they are playing a ball-tossing game with two other sex-matched participants who are also participating in the study at other university hospitals. After the participant has received the ball 50% of the time from each player for 2 min (baseline period), they are partially excluded by one co-player, the excluder, who only gives the participant 5% of their ball tosses. For this study, only the immediate behavioral reaction, i.e., the PP in the first 2 min of partial exclusion, was considered because our previous studies showed that HC immediately increase their PP for the excluding player during this period^4,20^. In that study, PP was different in both PDD and BPD patients in that they showed a less pronounced increase of PP towards the excluder; this difference was significant between PDD patients and HC but not between BPD patients and HC^20^. In brief, increased ball tosses towards the excluding player where are recorded as positive scores and those to the including player, as negative scores; a detailed description of how PP is calculated is provided elsewhere^20^.

### Statistical analyses

Analyses were performed with SPSS Statistics (Version 28) with a fixed significance threshold of *p* < 0.05. Group differences were calculated with one-way analysis of variance (ANOVA) for planned contrasts (HC vs. patients, BPD vs. HC_BPD_, PDD vs. HC_PDD_, BPD vs. PDD, HC_BPD_ vs. HC_PDD_). The Welch ANOVA test was used in case of a significant Levene’s test. Effect sizes were calculated according to Cohen^58^, with |0.1| being a small, |0.3| a medium, and |0.5| a large effect. To control for multiple testing, we used the FDR^59^. Pearson correlation with bias-corrected and accelerated bootstrap confidence intervals (BCa 95% CI), with 1000 bootstrap samples was used to test for correlations between the TAQ, CD-RISC-10, PP, and age. We conducted conditional process analyses using PROCESS macro and model 59^60^ to test the mediating effect of resilience on the relationship between adaptive and adverse life events on PP and the moderating effect of group (patient vs HC). Models were calculated for each adverse and adaptive life event subscale and each developmental phase of the TAQ and for the total scores (see Table S1). Confidence intervals calculated from 10,000 bootstrap iterations with 95% CI were used to control for significance of the indirect effect. Age was included as a covariate in the conditional process analyses because it may influence resilience^23^. Assumptions of linearity, homoscedasticity, and normality were checked. The independence of error terms was given (Durbin-Watson *d* = 1.70–1.79). Apart from the relationship between adverse events during childhood and adulthood in the TAQ and the CD-RISC-10, the relationships between almost all the TAQ subscales and the CD-RISC-10 were of some concern considering linearity and the independence of errors (Durbin-Watson *d* = 0.79–1.01). The proposed model is illustrated in Fig. S2 in the supplemental materials.

### Supplementary Information

Supplementary Information.

## Data Availability

Data will be made available on request. Please contact Barbara.Barton@med.uni-muenchen.de.
