# Supplementary material for: Effects of resilience and timing of adverse and adaptive experiences on interpersonal behavior: a transdiagnostic study in a clinical sample
Source: Sci Rep. 2023 Oct 24;13:18131. doi: 10.1038/s41598-023-44555-z (PMC10598007; doi:10.1038/s41598-023-44555-z)
Supplement: Supplementary file 1 — Supplementary Information. [file 41598_2023_44555_MOESM1_ESM.docx]

Figure S1

Scatterplots of the main findings


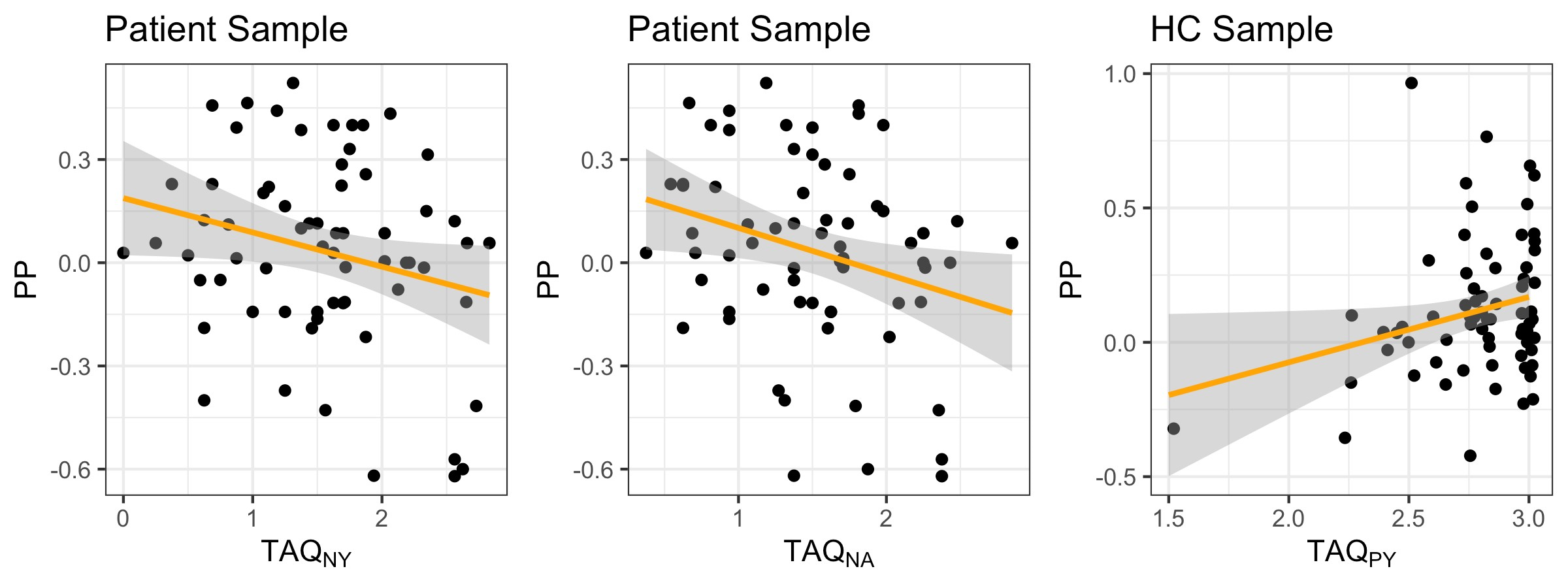


Note. PP: Passing Preference, HC = healthy controls, TAQ: Traumatic Antecedents Questionnaire, NY: negative life events during youth, NA: Negative life events during adulthood, PY: positive life events during youth

Figure S2

*Proposed model*

*X*: Adverse/Adaptive life events (TAQ)

*Y*: Passing preference (PP)

*M*: Resilience

(CD-RISC-10)

covariate: age

*W*: Diagnosis

BPD, PDD, HC_BPD_, HC_PDD_

*Note.* Mediation model of the effect of adverse and adaptive life events during childhood, youth and adulthood measured with the Traumatic Antecedents Questionnaire (TAQ) on passing preference (PP) during Cyberball through resilience measured with the Connor-Davidson Resilience Questionnaire (CD-RISC-10); HC, healthy controls: BPD, borderline personality disorder; PDD, persistent depressive disorder; *X*, independent variable; *M*, mediating variable; *W*, moderating variable; *Y*, dependent variable

Table S1

*Conditional Process Analyses with Age as a Covariate*

|  | Resilience, CD-RISC-10 (*M*) | Passing Preference, PP (*Y*) |
| --- | --- | --- |
| **Predictor** | Coefficients (*SE*) | Coefficients (*SE*) |
| TAQ_N_ (*X*) | -.712 (1.24), *p* = .566 | .05 (.05), *p* = .328 |
| Group (*W*) | -12.47 (2.40), ***p* < .001** | .35 (.22), *p* = .103 |
| Resilience (*M*) | - | .02 (.01), *p* = .193 |
| **Interaction term** |  |  |
| *X* × *W* | *R*^2^ = .00, *F* (1,130) = 0.02, *p* = .885 | *R*^2^ = .02, *F* (1,128) = 2.21, *p* = .140 |
| *M* × *W* |  | *R*^2^ = .03, *F* (1,128) = 3.69, *p* = .057 |
|  | **Effects (*SE*)** | ***95% CI*** |
| **Conditional direct effects** |  |  |
| Healthy Controls | .01 (.03), *p* = .797 | -.044; .058 |
| Patients | -.04 (.018), ***p* = .031** | -.077; -.004 |
| **Conditional indirect effects** |  |  |
| Healthy Controls | -.001 | -.011; .008 |
| Patients | -.007 | -.007; .023 |
| Index of moderated mediation | .008 | -.009; .026 |
| TAQ_NC_ (*X*) | -4.27 (3.31), *p* = .199 | .21 (.15), *p* = .168 |
| Group (*W*) | -13.19 (1.83), ***p* < .001** | .28 (.21), *p* = .180 |
| Resilience (*M*) | - | .02 (.01), *p* = .172 |
| **Interaction term** |  |  |
| *X* × *W* | *R*^2^ = .00, *F* (1,130) = 0.63, *p* = .427 | *R*^2^ = .02, *F* (1,128) = 2.28, *p* = .133 |
| *M* × *W* |  | *R*^2^ = .03, *F* (1,128) = 3.60, *p* = .060 |
|  | **Effect (*SE*)** | ***95% CI*** |
| **Conditional direct effects** |  |  |
| Healthy Controls | .08 (.07), *p* = .259 | -.061; .223 |
| Patients | -.04 (.04), *p* = .299 | -.130; .040 |
| **Conditional indirect effects** |  |  |
| Healthy Controls | -.01 | -.050; .022 |
| Patients | .02 | -.013; .049 |
| Index of moderated mediation | .022 | -.018; .074 |
| TAQ_NY_ (*X*) | -.85 (3.26) *p* = .795 | .08 (.14), *p* = .575 |
| Group (*W*) | -12.33 (2.25) ***p* < .001** | .33 (.21), *p* = .117 |
| Resilience (*M*) | - | .02 (.01), *p* = .200 |
| **Interaction term** |  |  |
| *X* × *W* | R^2^ = .00, *F* (1,130) = 0.01, *p* = .933 | *R*^2^ = .10, *F* (1,128) = 1.45, *p* = .231 |
| *M* × *W* |  | *R*^2^ = .26, *F* (1,128) = 3.67, *p* = .058 |
|  | **Effect (*SE*)** | ***95% CI*** |
| **Conditional direct effects** |  |  |
| Healthy Controls | -.02 (.07), *p* = .772 | -.150; .112 |
| Patients | -.12 (.05), ***p* = .016** | -.213; -.023 |
| **Conditional indirect effects** |  |  |
| Healthy Controls | -.00 | -.025; .019 |
| Patients | .02 | -.021; .054 |
| Index of moderated mediation | .02 | -.026; .059 |
| TAQ_NA_ (*X*) | .15 (3.32) *p* = .964 | .12 (.14), *p* = .396 |
| Group (*W*) | -12.38 (2.54) ***p* < .001** | .35 (.21), *p* = .095 |
| Resilience (*M*) | - | .01 (.01), *p* = .221 |
| **Interaction term** |  |  |
| *X* × *W* | R^2^ = .00, *F* (1,131) = 0.06, *p* = .805 | R^2^ = .02, *F* (1,129) = 2.35, *p* = .128 |
| *M* × *W* |  | R^2^ = .02, *F* (1,129) = 3.37, *p* = .069 |
|  | **Effect (*SE*)** | ***95% CI*** |
| **Conditional direct effects** |  |  |
| Healthy Controls | -.01 (.07), *p* = .869 | -.141; .119 |
| Patients | -.14 (.06), ***p* = .013** | -.255; -.031 |
| **Conditional indirect effects** |  |  |
| Healthy Controls | -.00 | -.021; .017 |
| Patients | .01 | -.027; .053 |
| Index of moderated mediation | .01 | -.029; .057 |

Table S1 continued

|  | Resilience, CD-RISC-10 (*M*) | Passing Preference, PP (*Y*) |
| --- | --- | --- |
| **Predictor** | Coefficients (*SE*) | Coefficients (*SE*) |
| TAQ_P_ (*X*) | 4.65 (2.28) ***p* = .044** | .20 (.11) *p* = .067 |
| Group (*W*) | 4.99 (9.84) *p* = .613 | .87 (.45) *p* = .053 |
| Resilience (*M*) | - | .01 (.01) *p* = .528 |
| **Interaction term** |  |  |
| *X* × *W* | R^2^ = .01, *F* (1,130) = 2.85, *p* = .094 | R^2^ = .02, *F* (1,128) = 2.95, *p* = .088 |
| *M* × *W* |  | R^2^ = .01, *F* (1,128) = 1.66, *p* = .200 |
|  | **Effect (*SE*)** | ***95% CI*** |
| **Conditional direct effects** |  |  |
| Healthy Controls | .10 (.05), *p* = .054 | -.002; .208 |
| Patients | .01 (.02), *p* = .607 | -.023; .039 |
| **Conditional indirect effects** |  |  |
| Healthy Controls | -.01 | -.060; .015 |
| Patients | -.01 | -.026; .002 |
| Index of moderated mediation | -.00 | -.029; .052 |
| TAQ_PC_ (*X*) | 6.21 (5.61) *p* = .271 | .36 (.25) *p* = .155 |
| Group (*W*) | -5.51 (8.08) *p* = .505 | .60 (.38) *p* = .114 |
| Resilience (*M*) | - | .01 (.01) *p* = .345 |
| **Interaction term** |  |  |
| *X* × *W* | R^2^ = .00, *F* (1,130) = 0.73, *p* = .395 | R^2^ = .01, *F* (1,128) = 1.61, *p* = .208 |
| *M* × *W* |  | R^2^ = .02, *F* (1,128) = 2.47, *p* = .118 |
|  | **Effect (*SE*)** | ***95% CI*** |
| **Conditional direct effects** |  |  |
| Healthy Controls | .20 (.12), *p* = .118 | -.051; .444 |
| Patients | .03 (.03), *p* = .330 | -.033; .098 |
| **Conditional indirect effects** |  |  |
| Healthy Controls | -.00 | -.068; .031 |
| Patients | -.02 | -.053; .004 |
| Index of moderated mediation | -.01 | -.061; .053 |
| TAQ_PY_ (*X*) | 5.47 (5.55) *p* = .326 | .46 (.25) *p* = .063 |
| Group (*W*) | -6.48 (7.88) *p* = .413 | .72 (.37) *p* = .053 |
| Resilience (*M*) | - | .01 (.01) *p* = .346 |
| **Interaction term** |  |  |
| *X* × *W* | R^2^ = .00, *F* (1,130) = 0.59, *p* = .443 | R^2^ = .02, *F* (1,128) = 2.73, *p* = .101 |
| *M* × *W* |  | R^2^ = .02, *F* (1,128) = 2.54, *p* = .114 |
|  | **Effect (*SE*)** | ***95% CI*** |
| **Conditional direct effects** |  |  |
| Healthy Controls | .25 (.12), ***p* = .041** | .011; .490 |
| Patients | .04 (.03), *p* = .209 | -.024; .110 |
| **Conditional indirect effects** |  |  |
| Healthy Controls | -.00 | -.071; .031 |
| Patients | -.01 | -.046; .005 |
| Index of moderated mediation | -.01 | -.056; .060 |
| TAQ_PA_ (*X*) | 13.55 (5.23) ***p* = .011** | .28 (.25) *p* = .257 |
| Group (*W*) | 5.19 (7.64) *p* = .498 | .50 (.35) *p* = .164 |
| Resilience (*M*) | - | .01 (.01) *p* = .501 |
| **Interaction term** |  |  |
| *X* × *W* | R^2^ = .02, *F* (1,131) = 5.18, ***p* = .024** | R^2^ = .01, *F* (1,129) = 1.71, *p* = .193 |
| *M* × *W* | - | R^2^ = .01, *F* (1,129) = 1.48, *p* = .226 |
|  | **Effect (*SE*)** | ***95% CI*** |
| **Conditional direct effects** |  |  |
| Healthy Controls | .11 (.12), *p* = .355 | -.129; .358 |
| Patients | -.06 (.04), *p* = .178 | -.136; .025 |
| **Conditional indirect effects** |  |  |
| Healthy Controls | -.01 | -.117; .068 |
| Patients | -.01 | -.044; .018 |
| Index of moderated mediation | -.00 | -.083; .109 |

*Note.* *X*, independent variable; *M*, mediating variable; *W*, moderating variable; *Y*, dependent variable; PP, passing preference during social exclusion (higher PP scores indicate increased ball tosses towards the excluder [4, 20]);

TAQ, Traumatic Antecedents Questionnaire, TAQ_N_, total score negative life events; TAQ_NA_, adverse events during adulthood; TAQ_NC_, adverse events during childhood; TAQ_NY_, adverse events during youth; TAQ_P_, total score adaptive life events; TAQ_PA_, adaptive events during adulthood; TAQ_PC_, adaptive events during childhood; TAQ_PY_, adaptive events during youth

during youth, TAQ_PA_ = adaptive events during adulthood,

*α* = .05, significant results are indicated in bold face

*Specific Note.* The TAQ was completed by 35 patients with BPD, 31 patients with PDD, 36 HC_BPD_, and 33 HC_PDD_; the CD-RISC-10 was completed by 36 patients with BPD, 33 patients with PDD, 36 HC_BPD_, and 34 HC_PDD_.
